# Correction: Implementing the IMPALA continuous monitoring system for paediatric critical care in Malawi: A mixed methods study of barriers and facilitators

**DOI:** 10.1371/journal.pone.0339889

**Published:** 2025-12-30

**Authors:** Daniel Mwale, Christopher Pell, Jobiba Chinkhumba, Happiness Mandala, Jessica Chikwana, Josephine Langton, Alice Likumbo, Michael Boele van Hensbroek, IMPALA Study Team, Job Calis, Wendy Janssens, Lucinda Manda-Taylor

In the Results subsection of the Abstract, there is an error in the second sentence. The correct sentence is: Healthcare providers reported spending less time on child monitoring after the introduction of IMPALA (3.3 hours before 95% CI: 2.36–4.23), compared to 1.8 hours per day after introducing IMPALA (95% CI: 1.19–2.48, p < 0.001).
