# Supplementary material for: Implementing the IMPALA continuous monitoring system for paediatric critical care in Malawi: A mixed methods study of barriers and facilitators
Source: PLoS One. 2025 Aug 12;20(8):e0329265. doi: 10.1371/journal.pone.0329265 (PMC12342236; doi:10.1371/journal.pone.0329265)
Supplement: S1 File — (DOCX) [file pone.0329265.s004.docx]

**Annex 1: Acknowledgements**

**The IMPALA Study Team†**

**Group Lead: Job Calis**

**Email:** [**impala.edctp@gmail.com**](mailto:impala.edctp@gmail.com)

Collaborators in the IMPALA study team include: Job Calis MD PhD, Amsterdam Institute for Global Health and Development, Amsterdam, The Netherlands; Christopher Pell PhD, Amsterdam Institute for Global Health and Development, Amsterdam, The Netherlands; Wendy Janssens PhD Prof, Amsterdam Institute for Global Health and Development, The Netherlands; Ângela Jornada Ben MD PhD, Amsterdam Institute for Global Health and Development, The Netherlands; Daniella Brals PhD, Amsterdam Institute for Global Health and Development,The Netherlands; Mark Hoogendoorn PhD Prof, Amsterdam Institute for Global Health and Development, The Netherlands; Michaël Boele van Hensbroek MD PhD Prof, Amsterdam Institute for Global Health and Development,The Netherlands; Job van Woensel MD PhD Prof, Emma Childrens’ Hospital of the Amsterdam University Medical Centers, The Netherlands; Natacha Berbers MSc, Amsterdam Institute for Global Health and Development, The Netherlands; Nina Meels MSc, Amsterdam Institute for Global Health and Development, The Netherlands; Valeria Cristofoli MSc, Amsterdam Institute for Global Health and Development, The Netherlands; Keerthana Raghavan MSc, Amsterdam Institute for Global Health and Development, The Netherlands; Niek Versteegde MD, GOAL 3 B.V., ’s-Hertogenbosch, The Netherlands; Bart Bierling MSc, GOAL 3 B.V., ’s-Hertogenbosch, The Netherlands; Eline Pieck-KleinJan MSc, GOAL 3 B.V., ’s-Hertogenbosch, The Netherlands; Eveline Geubbels PhD, GOAL 3 B.V., ’s-Hertogenbosch, The Netherlands; Lieke de Mare MSc, GOAL 3 B.V., ’s-Hertogenbosch, The Netherlands; Lennart Blom MD PhD, GOAL 3 B.V., ’s-Hertogenbosch, The Netherlands; Michael Levin MD PhD Prof, Section of Paediatric Infectious Disease, Imperial College London, London W2 1PG, UK; Aubrey Cunnington MD PhD, Section of Paediatric Infectious Disease, Imperial College London, London W2 1PG, UK; Myrsini Kaforou PhD; Section of Paediatric Infectious Disease, Imperial College London, London W2 1PG, UK; Clare Wilson MD, Section of Paediatric Infectious Disease, Imperial College London, London W2 1PG, UK; Diego Estrada-Rivadeneyra PhD, Section of Paediatric Infectious Disease, Imperial College London, London W2 1PG, UK; Shea Hamilton PhD, Section of Paediatric Infectious Disease, Imperial College London, London W2 1PG, UK; Victoria Wright PhD, Section of Paediatric Infectious Disease, Imperial College London, London W2 1PG, UK; Jonathan Sturgeon PhD, Section of Paediatric Infectious Disease, Imperial College London, Norfolk Place, London W2 1PG, UK; Grieves Mang’anda Jr MPH, Kamuzu University of Health Sciences, Malawi; Marrianne Kasiya MPH, Kamuzu University of Health Sciences, Malawi; Arox W. Kamng’ona PhD Prof, Kamuzu University of Health Sciences, School of Life Sciences and Allied Health Professions, Blantyre, Malawi; Daniel Mwale MSc, Kamuzu University of Health Sciences, Malawi; David Chaima PhD, Kamuzu University of Health Sciences, Malawi; Jenala Njirammadzi- Maleta MD, Kamuzu University of Health Sciences, Malawi; Josephine Langton MBChB Ass Prof, Kamuzu University of Health Sciences, Malawi; Jacquline Msefula MSc, Kamuzu University of Health Sciences, Malawi; James Makina MD, Kamuzu University of Health Sciences, Malawi; Jobiba Chinkhumba PhD, Kamuzu University of Health Sciences, Malawi; Lucinda Manda Taylor PhD, Ass Prof Kamuzu University of Health Sciences, Malawi; William Nkhono MSc, Kamuzu University of Health Sciences, Malawi; Margret Havara BSc, Kamuzu University of Health Sciences, Malawi; Alick Vweza PhD, Malawi University of Business and Applied Sciences, Malawi; Brenald Dzonzi BSc, Malawi University of Business and Applied Sciences, Malawi; Chimwemwe Msosa PhD, Malawi University of Business and Applied Sciences, Malawi; Christina Chiziwa BSc, Malawi University of Business and Applied Sciences, Malawi; Lezzie Chirambo BSc, Malawi University of Business and Applied Sciences, Malawi; Theresa Mkandawire PhD Prof, Malawi University of Business and Applied Sciences, Malawi; María Villalobos-Quesada PhD, National eHealth Living Lab, Public Health and Primary Care Department, Leiden University Medical Center, The Netherlands; Margot Rakers MD, National eHealth Living Lab, Public Health and Primary Care Department, Leiden University Medical Center, The Netherlands; Foteini Klapsaki Bach, National eHealth Living Lab, Public Health and Primary Care Department, Leiden University Medical Center, The Netherlands; Kamija Phiri MD PhD Prof, Training Research Unit of Excellence, Malawi; Alice Likumbo BSc, Training Research Unit of Excellence, Malawi; Jessica Chikwana MD, Department of Paediatrics, Zomba Central Hospital, Malawi; Glory Mzembe MD, Training Research Unit of Excellence, Malawi; Mary Magoya MSc, Training Research Unit of Excellence, Malawi; Timothy Rambiki MD, Training Research Unit of Excellence, Malawi; Martin Mwangi PhD, Training Research Unit of Excellence, Malawi; Owen Mtambo PhD, Training Research Unit of Excellence, Malawi; Christopher Nkhata, Training Research Unit of Excellence, Malawi; Patrick Chalira, Training Research Unit of Excellence, Malawi.
