# Supplementary material for: Implementing the IMPALA continuous monitoring system for paediatric critical care in Malawi: A mixed methods study of barriers and facilitators
Source: PLoS One. 2025 Aug 12;20(8):e0329265. doi: 10.1371/journal.pone.0329265 (PMC12342236; doi:10.1371/journal.pone.0329265)
Supplement: Attachment — Submitted filename: Response to Reviewers.docx [file pone.0329265.s005.docx]

**Detailed responses to Reviewer 1’s comments**

*Reviewer 1 comments: Please clarify the sampling strategy for caregivers and healthcare providers to ensure transparency and highlight the representativeness of the study population.*

Response: Thank you for the suggestion, we have clarified the sampling strategy and included it in the methods section “Respondents sampling and recruitment”. Specifically for the qualitative data, the plan was as follows:

In total, 23 observations and in-depth interviews (IDIs) were conducted at Zomba Central Hospital's paediatric HDU, with healthcare providers (n = 14) and caregivers (n = 9) of nine critically ill children (aged 3 months to 5 years old) admitted to the HDU. After introducing the IMPALA monitors, study participants (healthcare providers and caregivers) were conveniently recruited for structured observations and in-depth interviews between November 9, 2022, and October 30, 2023. Healthcare providers of different cadres (nurses, clinical officers and medical doctors) working in HDU were recruited (face-to-face) during their day shifts, representing a mix of professional roles and levels of experience. Selection was based on their involvement in paediatric care, treatment and management. To enhance diversity and representativeness, caregivers of critically ill children with a range of diagnoses and socioeconomic status were also recruited during their admission to HDU. (Refer to lines 223-234)

Caregivers were selected based on being above eighteen years of age and fulfilling a caregiving role. This approach ensured capturing the lived experience of providing care for critically ill children (refer to lines 240-242)

For the quantitative data, the strategy was as follows:

The sampling frame for the questionnaire-based caregiver survey consisted of all caregivers of critically ill children aged three months to five years who were admitted to the HDU between January 1 and June 30, 2023, and consented to participate in the IMPALA clinical observational study. (Refer to lines 247 to 250)

Caregivers whose child died in HDU did not participate in the surveys. A maximum of 110 caregivers were expected to be interviewed, dependent on the number of admissions and caregiver consent during the data collection period. (Refer to lines 256 to 258)

The sampling frame for the questionnaire-based healthcare provider survey comprised all healthcare providers (i.e., the complete census of nurses and clinicians) working in the HDU from January to May 2023. (Refer to lines 258 to 261)

*Reviewer 1 comments: Consider discussing potential selection and courtesy bias in caregiver responses, especially since interviews were conducted post discharge.*

Response: Thank you for bringing this point to our attention. We have added a paragraph describing how selection and interviewer bias were addressed in the section “Strengths and limitations of the study”, as follows:

We noted that interviewing caregivers after discharge may have introduced selection bias, as those with either more positive or more negative experiences could lead to an increased willingness to participate. However, all the invited caregivers consented to participate, such that selection bias remained limited. (Refer to lines 963 to 966)

Observer effects may also have influenced participants’ behaviour during the HDU observations. While researchers made efforts to minimise disruption and blend into the clinical environment, their presence may still have affected how staff interacted with the monitors. Additionally, caregiver responses may have been influenced by courtesy bias, particularly if gratitude towards healthcare providers shaped their feedback. To mitigate this, participants were assured at the start of each interview that their responses would remain anonymous and would not affect their future access to care. (Refer to lines 968 to 974.)

*Reviewer 1 comments: Clarity and language: thorough language review is recommended to correct grammatical inconsistencies, repetitive phrasing, and tense shifts.*

Response: Thank you for your comment. This has been addressed by reading and re-reading the manuscript, and making corrections accordingly.

*Reviewer 1 comments: Please revise long or redundant sentences, particularly in the discussion section, to improve readability.*

Response: Thank you for bringing this important point to our attention. The discussion has been double-checked for long and redundant formulations, and revised accordingly. (Refer to lines 793 to 941).

*Reviewer 1 comment: Ethical procedure: Kindly specify who conducted the informed consent process and how it was administered, especially for illiterate participants.*

Response: Thank you for your suggestion; we have included statements in the section “ethical consideration” to address the issue raised, as follows (Refer to lines 361 to 368)

Written informed consent, translated into the local language, was provided to all participants by DM, including caregivers of critically ill children and healthcare providers, for observations, in-depth interviews, and questionnaire-based surveys, with a signature or thumbprint. In cases of illiterate participants, DM read the information sheet and written consent form aloud in their local language. These participants were then asked to confirm their understanding, provide verbal consent voluntarily, and provide a thumbprint.

*Reviewer 1 comment: Data Availability Statement: The current statement indicates that data are available upon request due to confidentiality. In line with PLOS ONE's open data policy, I recommend that the authors de-identify and publicly share applicable quantitative datasets or provide a clear justification for the restriction.*

Response: The quantitative data set has been de-identified and will be publicly shared per the journals’ requirements.

*Reviewer 1 comment: Statistical analysis: Where appropriate, please consider including confidence intervals or p-values to give more context to the descriptive statistics*.

Response: Thank you for your suggestion. We have taken it on board and added confidence intervals to Tables 1 and 2, adjusting the text accordingly. (Refer to lines 452-465 for the discussion of table 1 results and to lines 518-525 for table 2 results).

*Reviewers 1 comment: Clarify whether any comparative analyses were performed (e.g., before vs. after implementation, or between trained vs. untrained staff), even if exploratory.*

Response: Thank you for your valuable comment. We have carefully considered this and included it in the section “Results: Impact on the workload of healthcare providers”, as follows:

The healthcare provider questionnaire-based surveys echo this finding. On average, staff reported spending 1.8 hours (95% CI: 1.19-2.48) on child monitoring on a typical day during the IMPALA implementation pilot, compared to 3.3 hours (95% CI: 2.36-4.23) before the implementation of IMPALA, a statistically significant difference (p-value < 0.001). (Refer to lines 515-518).

The Methods sub-section “Data processing and analysis” has been updated in line, as follows:

The analyses include inferential statistics (95% confidence intervals) for all variables, as well as comparative statistics for the before-after difference in hours worked on child monitoring. (Refer to lines 352-354)

*Reviewer 1 comment: Policy implications and scalability: consider expanding the conclusion to highlight how these findings can inform broader implementation or scale-up in other low-resource settings.*

Response: Thank you for your important suggestion. We have expanded the conclusion to inform the broader implementation in the section “Conclusion”, as follows (Refer to lines 1009 to 1037):

The IMPALA monitoring system has demonstrated significant value in improving the management of critically ill children at Zomba Central Hospital. Its ability to simultaneously and continuously monitor multiple vital signs—heart rate, respiratory rate, blood pressure, and oxygen saturation—was viewed favourably by both healthcare providers and caregivers. Compared to intermittent monitoring methods, continuous monitoring was perceived as more reliable, less labour-intensive, and better suited to support timely clinical decision-making.

For long-term adoption and potential scale-up of IMPALA in other low-resource settings, several areas for improvement were identified. These include enhancing battery capacity to ensure functionality during frequent power outages, optimising the user interface through ongoing caregiver and provider input, and reducing alarm fatigue through improved alarm management strategies. Ongoing, structured training for healthcare providers is also essential to ensure consistent and competent use of the device.

Successful integration into routine clinical workflows will depend not only on technological improvements but also on broader health system strengthening. Embedding IMPALA within existing critical care protocols, supported by targeted training in paediatric monitoring and clinical response, will be critical for sustained use. Equally important is addressing caregiver trust and misconceptions, particularly fears linking monitoring devices to child mortality. Promoting transparent communication, clarifying caregivers’ roles, and actively engaging them as partners in care can foster trust, reduce anxiety, and alleviate the burden on overstretched clinical staff.

More broadly, as continuous monitoring technologies become increasingly viable for LMICs, these findings offer practical guidance for policymakers, programme implementers, and health system planners. Lessons from this implementation study can inform procurement strategies, integration plans, and community engagement approaches across similar settings. Tailoring implementation to local health system capacity, workforce constraints, and cultural context will be essential to ensuring sustainable adoption and ultimately improving paediatric critical care outcomes across low-resource environments.

*Reviewer 1 comment: Monitor design and alarm features: Given the recurring issue of false alarms and alarm fatigue, please elaborate on whether alarm thresholds or settings were adjustable and how this will be addressed in future iterations of the IMPALA monitor.*

Response: Thank you for your comment. We now provide further information on the adjustability of the alarms. (Refer to lines 142 to 144)

Unlike conventional systems, IMPALA features a novel, context-specific design with adjustable alarms, configurable settings for end-users, a scroll-based menu, and a simplified interface.

*Reviewer 1 comment: Visual Aids and Figures: Please ensure that all figures (e.g., floor plan, monitor diagram) are of publication quality, with clear labelling and resolution.*

Response: Thank you for the suggestion. We have updated all our figures accordingly.

*Reviewer 1 comment: Non-response in Survey Data: In Table 1, nearly half of the caregivers did not respond to some items related to alarm response. Please address this non-response and discuss its potential implications for the interpretation of the data.*

Response: Your comment made us realise that the footnote in the Table 1 was not correct. The low number of alarm responses was not due to non-response but to a skip in the survey: If the alarm had not sounded during the child’s HDU stay, the caregiver was not asked about how the healthcare worker responded; instead, the question was skipped. We have now included a clearer footnote to the table and addressed this more adequately in the text in the section “Results”, as follows.

Over half, 51.3 % of the caregivers (95% CI: 39.6-63-2), always or sometimes heard an alarm sounding during the HDU stay. Among caregivers who had experienced an alarm sounding (n=38), 94.7% (95% CI: 87.3–100) said HCPs sometimes or always responded promptly, while 73.7% (95% CI: 59.0–88.4) reported that they “always” responded. (Refer to lines 461 to 465)

We have also added a reference to this in the Results subsection “Determinants of monitor use”, as follows:

Indeed, approximately half (49%) of the caregivers in the survey reported that they did not hear the alarms sounding during their child’s stay on the HDU (Table 1). During observations, it was also noted that the nurses changed the alarm thresholds to high or low numbers or turned off the alarm to minimise beeping. (Refer to lines 614 to 618)

**Response to Reviewer 2’s comments**

*Reviewer 2 comment:* *There are just a few minor language and typographical errors that can be addressed at the copyediting stage (e.g., a few spelling inconsistencies like “verson” instead of “version”). Otherwise, the manuscript is written in clear and standard English.*

Response: Thank you for your comment. We have re-read the manuscript to further eliminate any remaining spelling mistakes, and we have removed “version” in the section “Abstract under methods”. (Refer to lines 33 to 40).

**Detailed responses to Reviewer 3’s comments**

*Reviewer 3 comment: Introduction: The researchers are encouraged to add some statistics from the literature regarding morbidity/ mortality of children in critical care units in Malawi or other relevant healthcare contexts. This could further clarify the research problem*

Response: Thank you for this very useful suggestion. We have searched the literature and found statistics on peadiatric mortality in critical care units in three hospitals in Malawi, as well a meta-analysis for low- and middle-income countries. We have added these statistics in the first paragraph of the section “Introduction”. (Refer to lines 74 to 78).

Three studies conducted in various tertiary hospitals with HDUs in Malawi reported paediatric hospital mortality rates ranging from 2.2% to 4.4%, which aligns with the average paediatric hospital mortality rate of 4.1% reported in a recent meta-analysis of studies conducted in LMICs [4–7]. A major cause of in-hospital mortality is related to the late detection of deteriorating vital signs [8].

*Reviewer 3 comment: Method and setting: Design: The researchers are recommended to report the reason for selecting the mixed method design and explain how it was useful in investigating the research problem.*

Response: Thank you for raising an important point. We have added a statement in the section “Methods and Settings”. (Refer to lines 129 to 134):

A mixed-methods design was chosen for the following reasons: observations and interviews were conducted to mitigate any potential desirability bias that might arise from the observations. At the same time, surveys were conducted to examine and quantify the responses from the wider set of respondents. The triangulation of data sources enhanced methodological rigour and strengthened the validity of the findings.

*Reviewer 3 comment: Data collection: The researchers are encouraged to attach the two structured observation guides*

Response: Thank you for your comment. The two structured observation guides are attached as supplementary documents on the portal.

*Reviewer 3 comment: The questionnaire: The researchers are recommended to report information about the validity and reliability of the self-administered EMPATHIC-65 questionnaire.*

Response: Thank you for your meaningful feedback. Information on the validity of the self-administered EMPATHIC-65 questionnaire was already included during the initial submission in the section. As described in the initial submission, this existing questionnaire was subsequently adapted to the study context and translated from English into Chichewa. The questionnaire was expanded to encompass caregiver experiences during admission and vital signs monitoring (Refer to lines 307-309). We have now added to the manuscript that:

The revised questionnaire was extensively piloted among caregivers and further adjusted before data collection. (Refer to lines 313-314)

*Reviewer 3 Comment: Intervention: The researchers should add more information to support the novelty of the IMPALA Continuous Monitoring System.*

Response: Thank you for bringing this crucial point to our attention. We have expanded the section “Methods and setting: Intervention” as follows (Refer to lines 142 to 146):

Unlike conventional systems, IMPALA features a novel, context-specific design with adjustable alarms, configurable settings for end-users, a scroll-based menu, and a simplified interface. Its power-independent operation, enabled by up to four hours of battery backup, makes it uniquely suited for environments where electricity and infrastructure are often unreliable.

*Reviewer 3 comment: Quantitative Data: The researchers did not use inferential statistics. Such statistical tests could help to explain the credibility of the intervention.*

Response: Thank you for the important point, we have taken this on board and included inferential statistics (notably 95% confidence intervals) in Tables 1 and 2. In addition, we have added a statistical comparison in section “Results: Using the IMPALA monitor to support clinical decision-making” on the hours worked on monitoring before and after the implementation of IMPALA (Refer to lines 515 to 518)

On average, staff reported spending 1.8 hours (95% CI: 1.19-2.48) on child monitoring on a typical day during the IMPALA implementation pilot, compared to 3.3 hours (95% CI: 2.36-4.23) before the implementation of IMPALA, a statistically significant difference (p-value < 0.001).

***Reviewer 3 comment: Sample size****: The sample size is recommended to be calculated for the statistical test used to ensure the power, ES, and alpha level of significance are appropriate for the reported sample size.*

Response: Thank you for your suggestion, we have added a paragraph on sample size in the section “Methods and settings: questionnaire survey”. We note that both the sample of caregivers and sample of healthcare staff interviewed in the survey consisted of the entire census of caregivers and staff present during the data collection study period, who consented to be interviewed. The sample size calculation for the statistical comparison of hours worked on child monitoring before and after the introduction of IMPALA is included in the manuscript as follows:

We estimated the minimum detectable effect size of the change in hours spent on monitoring children before and after IMPALA for the sample of n=24 healthcare workers, based on a one-sample mean t-test power calculation with alpha=0.05, beta=0.80 and a standard deviation of 1.1025, to be 0.597. Though the sample size was small, it was appropriate for the exploratory, early-stage nature of this study. It was chosen to balance statistical power with feasibility in a resource-constrained setting. (Refer to lines 261 to 266).

*Reviewer 3 comment: Results: The quality of the figures should be improved regarding clarity and resolution.*

Response: Thank you for your observation and comment. We have improved and revised the figures accordingly. Please refer to the supplementary items.

**Detailed responses to Reviewer 4’s comments**

*Reviewer 4 comment: Abstract: The abstract is overloaded with numeric details (e.g., 300+ hours of observation, n=14, n=72) which detract from the clarity of the findings. The text would benefit from a more concise presentation using compact summaries, such as “Using over 300 hours of observations, interviews with 14 providers and 9 caregivers, and surveys from 24 providers and 72 caregivers…”*

Response: Thank you for your comment. We have revised the abstract accordingly. (Refer to lines 33 to 40).

A mixed-method implementation study of the IMPALA system in the paediatric High-dependency unit of a tertiary hospital from November 2022 to October 2023. Data were collected through over 300 hours of observations, in-depth interviews with 14 healthcare providers and nine caregivers of admitted children, and questionnaire-based surveys from 24 healthcare providers and 72 caregivers. Qualitative data were analysed thematically using inductive and deductive approaches. Descriptive statistics (frequencies, percentages, means, and standard deviations) were calculated for categorical and continuous variables.

*Reviewer 4 comment: Introduction: The introduction could benefit from a more technically specific description of the IMPALA system. For example, outlining the technical advantages, usability features, or target age group would help better position the device within the paediatric care landscape****.***

Response: Thank you for your important suggestion. We have considered this and added statements to the section “Introduction”. (Refer to lines 101 to 104)

IMPALA was developed using a user-centred design approach, incorporating the needs of nurses, doctors and clinical officers, such as visual alerts, adjustable alarm thresholds and simplified language. The device monitors vital signs, including heart rate, respiratory rate, oxygen saturation and blood pressure, in critically ill children aged between 28 days to 5 years.

*Reviewer 4 comment: Methods: The methods section is comprehensive and demonstrates strong methodological planning. The use of multiple data sources and triangulation is clear strength. However, the section is too lengthy and contains some redundancies. For example, the detailed training procedures on IMPALA use could be summarized and partially moved to the Results or Supplementary Materials.*

Response: Thank you for your comment. We included detailed training procedures on IMPALA use because they were part of the implementation process. This was useful for understanding how the device was implemented and examining the response of healthcare providers to the training they received.

*Reviewer 4 comment: References to figures (especially Figure 2) are underdeveloped. More context should be provided on how the physical setup of the HDU (e.g., multiple children sharing beds, limited equipment) influenced monitoring practices.*

Response: Thank you for your valuable suggestion. We have considered this and revised the figure. Refer to the supplementary item.

*Reviewer 4 comment: While the software used for analysis is clearly stated (NVivo, STATA), the manuscript should briefly describe how potential biases in qualitative interpretation (e.g., lack of blinding, interpreter bias) were mitigated.*

Response: Thank you for your critical comment. We have revised and included a paragraph that addresses how potential biases in qualitative interpretation were mitigated in the section “Study design”, as follows:

Observations and interviews were conducted to mitigate any potential desirability bias that might arise from the observations. At the same time, surveys were conducted to examine and quantify the responses from the wider set of respondents. The triangulation of data sources enhanced methodological rigour and strengthened the validity of the findings. (Refer to lines 130 to 134)

*Reviewer 4 Comment: Results: The results section is informative but overly long and repetitive. Several points (e.g., alarm features, technical issues) are discussed multiple times. The use of clear subheadings based on the CFIR domains is commendable but should be streamlined for brevity.*

Response: Thank you for your valuable comment. We have now added sub-headings based on the CFIR domain throughout the Results section where appropriate.

*Reviewer 4 comment: A significant theme—caregiver mistrust and superstition (e.g., “IMPALA kills children”)—is only superficially covered. Its prevalence, distribution by caregiver demographics, and potential impact on implementation should be further explored or quantified.*

**Response:** Thank you for your interesting question. We agree that this is an important theme. However, we have very limited data on its prevalence and distribution by caregiver demographics, as this was not asked in the quantitative survey. Therefore, we cannot further quantify its significance. It only came up in the qualitative IDIs.

According to our qualitative data, four out of nine of our female caregivers reported feeling suspicious about the device that connecting critically ill children to the monitor could worsen their condition or lead to death. We have added this to the text (Refer to lines 922 to 924)**.**
